# Ordered structure of the transcription network inherited from the yeast whole-genome duplication

**DOI:** 10.1186/1752-0509-4-77

**Published:** 2010-06-03

**Authors:** Diana Fusco, Luigi Grassi, Bruno Bassetti, Michele Caselle, Marco Cosentino Lagomarsino

**Affiliations:** 1 Università degli Studi di Milano, Dip. Fisica. Via Celoria 16, 20133 Milano, Italy; 2 Duke University, Program in Computational Biology and Bioinformatics, North Building, 470 Research Drive, Durham NC 27708, USA; 3 Università degli Studi di Torino, Dip. Fisica Teorica, Via Giuria 1, 10125 Torino, Italy; 4 I.N.F.N. Milano, Via Celoria 16, 20133 Milano, Italy; 5 I.N.F.N. Torino, Via Giuria 1, 10125 Torino, Italy; 6 Genomic Physics Group, FRE 3214 CNRS "Microorganism Genomics" and University Pierre et Marie Curie, 15 rue de l'École de Médecine, 75006, Paris, France

## Abstract

**Background:**

Gene duplication, a major evolutionary path to genomic innovation, can occur at the scale of an entire genome. One such "whole-genome duplication" (WGD) event among the Ascomycota fungi gave rise to genes with distinct biological properties compared to small-scale duplications.

**Results:**

We studied the evolution of transcriptional interactions of whole-genome duplicates, to understand how they are wired into the yeast regulatory system. Our work combines network analysis and modeling of the large-scale structure of the interactions stemming from the WGD.

**Conclusions:**

The results uncover the WGD as a major source for the evolution of a complex interconnected block of transcriptional pathways. The inheritance of interactions among WGD duplicates follows elementary "duplication subgraphs", relating ancestral interactions with newly formed ones. Duplication subgraphs are correlated with their neighbours and give rise to higher order circuits with two elementary properties: newly formed transcriptional pathways remain connected (paths are not broken), and are preferentially cross-connected with ancestral ones. The result is a coherent and connected "WGD-network", where duplication subgraphs are arranged in an astonishingly ordered configuration.

## Background

An organism can respond to internal and environmental cues by the coordinated activation of large sets of genes through transcriptional regulation. This process can be described as a complex "network" of interactions, connecting binding sites of regulatory proteins (transcription factors, TFs) to regulatory DNA regions of their target genes [1]. Achieving an understanding of the evolutionary forces that shape the architecture of this network is fundamental for contemporary biology, where large-scale functional genomic data are increasingly accessible experimentally.

In particular, gene duplication is among the most widespread mechanisms for evolutionary genomic innovations [2,3]. Duplication gives rise to the widespread existence of gene families, where members share a common ancestor. Gene duplication can occur with different functional consequences [4,5] at the scale of a single gene or a medium-sized genomic segment, but also of a whole genome [3,6-8]. Specifically, the rare but revolutionary events of whole-genome duplication (WGD) are believed to be very important for the transition to complex organisms [6,9]. Duplicate genes that persist in multiple copies may diverge by differentiation of sequence and function. This process is affected by factors including pathway redundancy and modularity, as well as dosage of gene expression.

As a consequence, gene duplication is also an important mechanism for the shaping of complex regulatory networks (which have to "manage" the repertoire of genes available to a genome) during evolutionary growth of a genome. The analysis of the currently available transcriptional network topologies from functional genomics experiments [10-13] has considerably increased our insight into network architecture and evolution, both on local and global scales. However, previous results have rarely been able to isolate *general rules*, which might potentially underly changes in the regulatory interactions after a duplication event, or which could describe the consequences of interaction changes on the fate of duplicate genes [14].

Here, we report a case where some general rules appear to emerge. We adopted a mathematical/physical approach to the systematic analysis of the topological properties of a transcription network after a WGD, considering the yeast *S. cerevisiae*. For this species, the transcription network topology is known and has been explored to a considerable extent [1]. Studies of comparative genomics demonstrated that *S. cerevisiae *arose from an ancient WGD [7,15-17], that occurred 100-150 million years ago. The evolutionary history of individual genes is well mapped [4,18]. Our method is based on elementary "duplication subgraphs", relating ancestral interactions with newly formed ones following duplications of known age.

## Methods

We used the set of transcriptional regulatory interactions of yeast compiled by Balaji and coworkers [11], consisting of4441 nodes (genes) and 12899 regulatory links assembled from the results of genetic, biochemical and ChlP-chip experiments.

Strictly speaking, in order to detect the fate of ancestral transcription factor-target interaction pairs after duplications, one would need data on the transcriptional network of the pre-duplication ancestor of *S.cerevisiae. *Clearly this data is not directly available. However, based on some simple assumptions, we can reconstruct an approximation of the pre-duplication network as a superposition of regulatory interactions among duplicated genes.

The standard way to perform this analysis [10,13,19] is to consider the distribution of interactions across homology classes, i.e., classes of network nodes which have a likely common ancestor. In our case, since we need to examine specifically the WGD and compare its effect to small-scale duplications, it is necessary to have additional information regarding the duplication date of paralogs. For this scope, we made use of the paralogue definitions from the Fungal Orthogroups Database v1.1 http://www.broadinstitute.org/regev/orthogroups/, which gives the orthogroup assignments for all predicted protein-coding genes across 23 Ascomycete fungal genomes. These data are built through a phylogenetic reconstruction of yeasts, and thus allow to estimate duplication dates [4]. More specifically, ref. [4] defines nine age groups divided into three pre-WGD, five post-WGD and a group of WGD duplicates. While the size of the duplication age groups varies greatly, this does not affect our study, as we consider normalized observables or scores against a null model. For most of our analyses, we regrouped the nine duplicate classes into post-WGD, WGD, and pre-WGD. Most of our attention was devoted to the pre-WGD to WGD transition. Overall, we considered 438 WGD pairs (of which 26 were TF pairs) and 363 non-WGD pairs (of which 28 involve TFs.)

**Figure 1:**
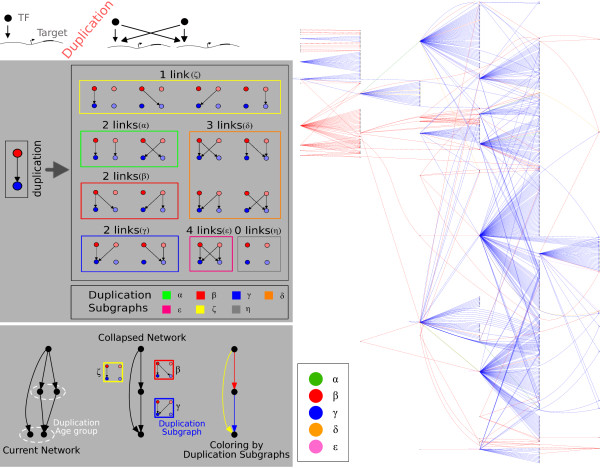
**The WGD transcription network of yeast**. Top-left panel: cartoon of the duplication-divergence model. Following a duplication, TF binding sites of duplicate promoters are initially identical. Evolution can retain memory of this initial condition, producing links in the network with consistent statistical patterns compared to homology classes [10,19]. Mid-left panel: scheme of all the possible DSs originating from an ancestral interaction. DSs are listed by number of links and node degree; DSs that are identical except for exchanges of the duplicate copies are grouped together, as 'old' and 'new' nodes cannot be distinguished in WGD. Each group is associated to a color and a greek letter. Bottom-left panel: scheme of the analysis procedure. First, the nodes of each duplication age class in the network and corresponding links are collapsed, in order to estimate the ancestral network. Subsequently, the DSs associated to each ancestral link to annotate the links. This operation is a coloring of the links, where each color corresponds to a DS. Fully disconnected *η*-type DSs are ignored. Right panel: the yeast "WGD network" of all nontrivial DSs (having two or more links). Although *ζ *type DSs, which make up 94.51% of the network, are not represented, the network is strikingly connected. Red (*β*, 1.1%) and blue (*γ*, 4.2%) DSs are heavily dominant. Furthermore, links of the same color are clustered and arranged in a hierarchical fashion (where red precedes blue). The occurrence of the other DSs is: *α*0.041%, *δ *0.049%, *ε *0.066%.

We estimated the ancestral networks by consecutive collapses of paralogous nodes inside the yeast transcription network (Fig. 1), adopting a conservative model where a link is considered to be present in the ancestral network each time an interaction is present between any of the collapsed nodes (for an estimate of link loss by rewiring of interactions see also [13]).

We analyzed the network structures emerging from each duplication age in terms of the "duplication subgraph" (DS) that can be defined to emerge from each link of the ancestral network (see Fig. 1). Duplication subgraphs can be visualized as colors on the edges of the ancestral (collapsed) network. Fig. 1 classifies DSs by symmetry, connectivity and number of links, assigning to each a color and a greek letter, in order to help in the discussion of the results.

## Results

The right panel of Fig. 1 summarises most of the results that will be quantified in the following. It represents the network formed by all duplication subgraphs for which at least one extra interaction has been inherited after the WGD (i.e. the DSs with at least two links, visualized as colors on the links of the ancestral network). We will refer to this structure as the "WGD network". This network includes only about 5.5% of the ancestral network links. It is essentially made up of a large single connected feedforward component, where DSs are arranged in an ordered and hierarchical configuration (note for example that red links tend to be upstream of blue ones). Fig. 1 classifies DSs in classes by link number and symmetry: *ζ *type DSs, with one link, make up 94.51% of the network, two-linked red (*β*, 1.1%) and blue (γ, 4.2%) DSs are heavily dominant on the rest. The occurrences of the other DSs are α 0.041%, δ 0.049%, ∈ 0.066%.

### Correlations of conserved interactions after duplications of TF-target pairs are conveniently represented in terms of Duplication Subgraphs

So far, we have introduced duplication subgraphs merely as a data structure. However, focusing on the pre-WGD to WGD transition, we have found that these entities are in some sense a necessary representation of the data. The simplest model for the retention of regulatory links after gene duplications assumes that the conservation of different links is independent [14,19]. However, while this approach can be successful in the case of (nondirected) protein interaction networks [14,20,21], this model of independent evolution appears insufficient to reproduce the empirical occurrence of the different WGD DSs. In terms of parameters, an independent model would contain at most four probabilities of link conservation (See Additional File 1, Section 1), but these probabilities and their products generally do not reproduce the empirical occurrence of DSs.

An alternative description that can be useful to adopt assigns conservation probabilities to edges *and *to the number of nodes conserved in a duplication subgraph. This is parametrically equivalent to the description in terms of duplication subgraphs, assuming that the nodes that remain isolated are eventually lost. In the following, we will mainly use the duplication subgraph framework, as it gives a simple view of correlations between adjacent duplicated interactions, useful for modeling the process.

### The duplication subgraphs stemming from the WGD are nontrivially distinct from those emerging from small-scale duplications

**Figure 2:**
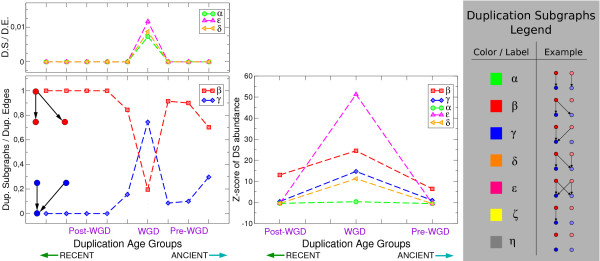
**The DSs stemming from the WGD are significantly different from those emerging from small-scale duplications**. Left panel: absolute abundance of DSs (normalized by the total number of duplicated edges, i.e. DSs with two or more links) plotted as a function of duplication age. For this analysis all the duplication age classes of ref. [4] were employed. We have plotted *α*-, *δ*- and *ε*- type DSs on a separate graph (upper left panel) because of the difference in scale. Central panel: plot of the relative DSs abundance with respect to a null model shuffling homology classes vs. duplication age. We considered the Z-score of DS abundance for the grouped pre-WGD, post-WGD and WGD duplication age classes. Both plots show that the trends of DS abundance are distinct for the WGD. Right panel: duplication subgraph legend.

On the other hand, the pattern of abundance of duplication subgraphs (and thus the necessity to represent data in terms of duplication subgraphs) is very specific of the WGD, and small-scale duplications behave quite differently. In order to show this, we compared the different duplication age classes, both in terms of the relative abundance of DSs, and in terms of their overrepresentation with respect to the standard random model that shuffles homology classes (without mixing transcription factors and targets [10]). Fig. 2 and Additional File 1, Tables S2 to S5 resume the results of the two analyses. Fig. 2 also contains a duplication subgraph legend that can be used as a reference to interpret most of the results that will follow.

The analysis of absolute abundance shows that the occurrence of every DS, normalized by the number of duplicated links, is WGD-specific. In particular, the WGD produced a larger number of *γ *subgraphs and of other DSs involving duplicate transcription factors. In terms of overrepresentation compared to the random model, all DSs are more enriched in the WGD than in pre- and post-WGD duplications. Considering the WGD and non-WGD sets collectively confirms this result (Additional File 1, Tables S2 to S5). This random null model compensates for the different number of duplicated TFs in the different duplication age groups, and in particular among WGD versus non-WGD duplicates. In particular, Additional File 1, Tables S2 to S5 show a higher statistical abundance of duplication subgraphs involving crosstalk between ancestral and newly-formed interactions involving WGD duplicates.

Note that all duplication subgraphs (and in particular the duplication subgraph of four links) are a construction that in the case of the WGD-produced duplications, corresponds to a single event where the TF and its target were duplicated at the same time. Outside the WGD, all the duplication subgraphs are accessible only to a series of successive small-scale duplications (belonging to the same age group in our analysis) of TFs and their targets. Thus, Fig 2 and Additional File 1, Tables S2 to S5 strictly compare the results of two different evolutionary processes, and show that they yield different results.

It has been empirically demonstrated [22] that the number of common regulatory inputs of paralogous proteins decays with age since the duplication event (measured by sequence similarity of paralogs). While the age resolution in our study is coarser and this observation is related to duplication subgraphs in a complex way, the finding can be reconciled with Fig. 2 by the fact that the fraction of *β*-type duplication subgraphs roughly decays with duplication age in non-WGD paralogs, when all post-WGD paralogs, which correspond to a short evolutionary time compared to the other age groups, are considered as one point (see Additional File 1, Table S1).

### One-shot duplication-divergence model based on duplication subgraphs

**Figure 3:**
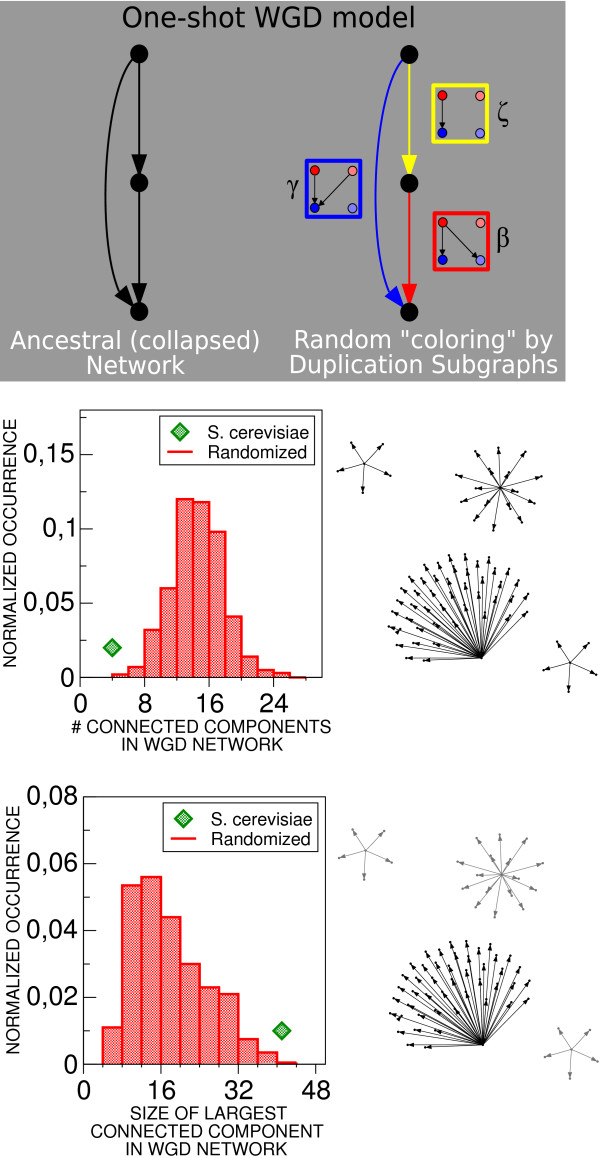
**One-shot WGD network duplication model and connected structure of the transcription-factor WGD network**. Top panel: in order to test for significant large-scale structures in the organization of duplication subgraphs in the network, we employed a one-shot network WGD (null) model, assigning to each ancestral edge a random duplication subgraph with probability equal to its empirical frequency. The model can be described as a random coloring of the ancestral (collapsed) network links. Middle and bottom panel: significance of the connectedness of the transcription-factor WGD network scored by the one-shot model. For each one of 4000 model realizations, we considered the histogram of the number of connected components (middle plot) and of the size of the largest connected component (bottom plot) of the network involving transcription-factor DSs with two or more links), and compared it to the empirical case. Networks containing a single large connected component as in the empirical one are very infrequent in the random model (P-value 0.001 for number, 5E-4 for size of connected components).

Fig. 1 suggests that DSs have an ordered configuration in the collapsed network representing the ancestral pre-WGD interactions. In other words, the clustered links of the same color and hierarchical distribution of red and blue links would be rarely observed if the coloring of these links were not correlated. In order to probe the significance of this observation, we designed a "one-shot" duplication-divergence model, where each link of the ancestral (collapsed) network is assigned a random duplication subgraph (i.e. is colored with prescribed probability). The basic entities of the model are thus individual duplication subgraphs, corresponding to ancestral regulatory interactions colored by the fate they followed during the WGD. This approach assumes a single collective duplication move as in a previous model by Presser and coworkers [14] and is not concerned with asymptotic properties as were other models present in the literature [20]. As illustrated in Fig. 3, this procedure is equivalent to a random coloring of the network links, where each color corresponds to a duplication subgraph. In particular, we assigned the empirical probabilities of producing each DS to the ancestral edges, so that randomized colorings would conserve the occurrence of each duplication subgraph.

Note that this model is very different from the standard null model shuffling homology classes that was used in the evaluation of DS occurrence [10,19]. The latter is designed to test the hypothesis that a given local network structure (a transcription factor -target interaction) stems from duplication, while the one-shot model probes the randomness of the distribution of duplication-inherited structures across the network, corresponding to colorings of the ancestral network.

### WGD duplication subgraphs form a connected network

The empirical WGD network, formed by all nontrivial DSs, is made of one large connected component and very few small ones. Using the one-shot duplication model, we considered the probability, in a random realization, that the WGD network formed by all nontrivial DSs has an equally dominant connected component (Fig. 3). We performed this test on the transcription factor WGD network (shown in Additional File 1, Figure S1), whose largest connected component is made of 41 (out of 48) DSs. This subnetwork constitutes the essential part of the WGD network, as only transcription factors can send out links. Our test indicates that the connectedness of the WGD network is significant (P-value 0.001 or less), which in fact justifies the notion of the WGD network itself.

### WGD duplication subgraphs have an uneven distribution in the hierarchical and feedback components of the network

**Figure 4:**
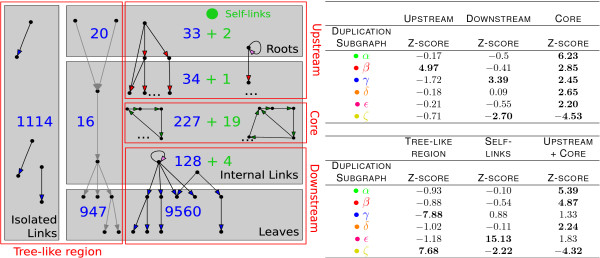
**Duplication subgraphs are organized hierarchically in the WGD network**. See the duplication subgraph legend in Fig. 2. Left panel: scheme of the hierarchical organization of the ancestral network's links. The numbers refer to the links found in each component. The "core" is the component formed by the set of links involved in feedback loops. The rest of the network is made up of feed-forward, tree-like components that are placed upstream or downstream of the core, or run "beside" it by connecting roots to leaves. Right panel: distribution of DSs in the hierarchical components of the network, scored by the WGD one-shot null model. The feedback core is enriched with all nontrivial DSs, the upstream part with *α *and *β *DSs, and the downstream part with *γ *DSs.

Duplication subgraphs of different kinds have different roles in the network. In order to show this, we divided the ancestral (collapsed) network into hierarchical components (see the cartoon in Fig. 4 and refs. [13,19]); we then estimated the enrichment of DSs in each component after the WGD with respect to our null model.

The central component is the feedback "core", i.e., the set of links involved in feedback loops, which we find to be enriched in all nontrivial DSs. The rest of the network is made up of feed-forward, tree-like components. The component upstream of the core (containing input nodes or "roots") is enriched in type *β *DSs, while the downstream component (containing target nodes or "leaves") is enriched in *γ *DS. Finally, the component running "beside" the core connecting roots to leaves is enriched in the trivial ζ-type DS. Interestingly, self regulators are enriched in ε DS, confirming previous results [13] obtained with a different model.

### Neighbouring duplication subgraphs are highly correlated

**Figure 5:**
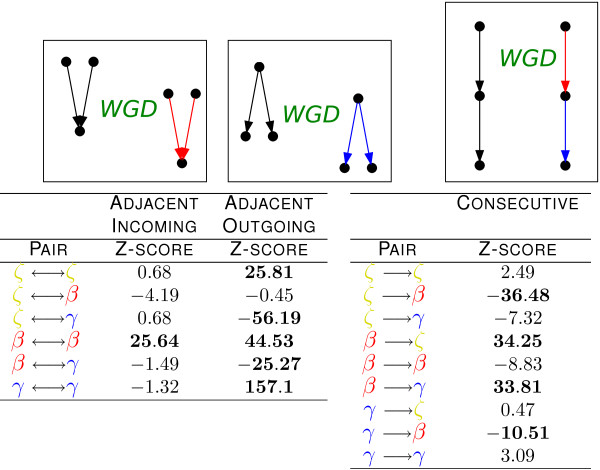
**Adjacent duplication subgraphs are correlated**. Neighbouring relationships between links in the ancestral network can be of three kinds: (i) two links emerge from the same node (adjacent outgoing), (ii) two links point to the same node (adjacent incoming), (iii) two links are arranged head to tail (consecutive). The tables contain correlation significance scores measured with the WGD one-shot model for the co-occurrence of neighbour DSs of type *ζ*, *β*, and *γ *in the ancestral network. The significantly co-occurring pairs indicate that adjacent outgoing links tend to generate equal DSs (see Additional File 1, Fig. S2.) Adjacent incoming links preferentially produce two γ DSs, and consecutive links are significantly enriched in *β *DSs upstream of *γ *subgraphs; both configurations maintain full crosstalk between ancestral and newly formed pathways. See the duplication subgraph legend in Fig. 2.

Finally, we used the one-shot model to probe the correlated occurrence of DSs of different types on the ancestral network (Fig 5). Neighbouring links in the ancestral network can be of three kinds: first, two links may emerge from the same node (adjacent outgoing). In this case, we find a marked preference for the same DS in both outgoing links, explaining the observed "patches" of the same color in the WGD network (Fig. 1). This correlation among adjacent outgoing links can be explained by individual duplicated transcription factors inheriting links which connect them to a correlated set of targets. Second, two links can point to the same node (adjacent incoming). Adjacent incoming links tend to be two *β *DSs, creating a feed-forward subgraph with four nodes and four links (resembling an ε-type DS). Interestingly, this pattern gives full cross-connectivity between the ancestral and the duplicate pathways. Third, two links can be arranged head to tail (consecutive). Many consecutive links consist of a *β *DSs upstream of a *γ*, again maintaining full cross-connectivity between ancestral and newly formed pathways. Moreover, this interaction displays a defined upstream-downstream hierarchy of DSs, which can also be observed in Fig. 1. We also tested explicitly whether the connectivity of pathways was maintained by the resulting DSs, or in other words whether transcriptional pathways could be broken after duplication, finding that connectivity is maintained with very high significance. (Additional File 1, Tables S6 to S8).

In summary, this analysis shows very characteristic patterns for neighbouring DSs, which are related to their observed ordered and hierarchical distribution within the network. We found significant enrichment of two *β *subgraphs for adjacent incoming interactions, of two *γ *DSs for adjacent outgoing links, and of *β – γ *pairs for consecutive interactions (see Additional File 1, Fig. S2 to visualize the interactions resulting from these correlations). This reflects an important underlying trend of WGD evolution, namely the preferential *cross-connectivity *of newly formed regulatory pathways with ancestral ones.

### General rules of whole transcription-network duplications, and links to spin models

The above result points to the question of whether at least some of the observed global ordering and hierarchical properties of WGD duplication subgraphs could emerge from the local correlation patterns of DSs. Biologically this would correspond to the hypothesis that the network organization can be seen by individual duplicate nodes only through their neighbours. From the modeling viewpoint, answering the above question requires transforming the one-shot network duplication model from a statistical tool to a positive (or predictive) model. In physical terms, the model is essentially a spin model, where spins, or colors representing DSs, are placed on links of the ancestral network.

While the one-shot null model is purely entropic, a variant can be introduced where the spins or colors interact with nearest neighbours through the network nodes (non-local interactions can also be considered, corresponding to different biological hypotheses). One major problem compared to conventional spin models is that there are three kinds of possible nearest neighbour interactions, corresponding to the possible arrangement of neighbouring links in the ancestral network. Notably, these interactions also correspond to three different effective interaction networks. For example, if only consecutive interactions are considered, the effective interaction network would treat all links connecting a root to a leaf as independent. For this reason, a simple spin model including consecutive interactions only cannot explain the empirical arrangement of DSs.

While many questions remain open concerning the inference of general duplication rules from this modeling approach, we have devised a simple model as proof of principle that the observed ordered behavior can be reproduced as the equilibrium state. In particular, the model considers only *ζ*, *β *and *γ *DSs, interacting through two coupling constants corresponding to sequential and adjacent interactions, plus two global external fields to constrain the magnetizations. Such a model, simulated with a straightforward Metropolis Monte Carlo algorithm, gives spin configurations that are much closer to the empirical observations compared to the uncoupled one-shot model; furthermore, the resulting equilibrium state shows the correct hierarchy and order (see text in Additional File 1, Section 5, and Additional File 1, Table S9).

## Discussion

We have shown that a WGD transcription network is a well-defined entity to study, as the network structure that emerges from this duplication event is connected and coherent. A simple quantitative picture of the process based on statistical independence of duplicated interactions is unsatisfactory in this context. Notably, the same independence hypothesis has proven to be successful in the context of protein interaction networks [14,20,21]. We can speculate that the reason for this could be a higher simplicity in protein-interaction with respect to transcriptional pathways. The failure of a simple model of independent link conservation obviously does not preclude the possibility that more sophisticated model variants using the hypothesis of independence (such as introducing an asymmetry between 'fast' versus 'slow' evolving nodes as in ref. [21]) might be useful to describe the system. On the other hand, the elements that form the WGD transcription network are very conveniently represented by duplication subgraphs, which can be visualized as colors on the edges of the ancestral network. The pattern of occurrence of DSs is hierarchical and highly correlated, and is distinct from the pattern seen for small-scale duplications.

The observation that DSs containing transcription factor nodes are more abundant and statistically enriched in the WGD is consistent with the previously reported abundance of WGD-duplicate transcription factors [23]. However, our findings go far beyond previous observations. First, we disentangle the multiplicity of possible structures formed by duplicate transcription factors. Second, we dissect location and distribution of the emergent WGD DSs. WGD duplication subgraphs are preferentially found in the network core, which is enriched for cell-cycle associated genes [13,24]; further, they are differentially distributed in the feed-forward regions upstream and downstream of the core. While a thorough functional analysis of the WGD transcription factor network was beyond our scopes, it could be useful to examine the regulatory pathways for all the known important functions developed by the WGD such as fermentation and anaerobic respiration in more detail.

The connectedness of transcriptional pathways emerging from the WGD, and the fact that DSs are found preferentially in the higher complexity regions of the ancestral network, could be related to the so-called "dosage-balance" hypothesis [4,23,25,26]. This hypothesis states that the only way to replicate a complex connected pathway would be to replicate it entirely, since any duplication of a single or a few nodes would at least initially render it unusable by creating an imbalance in the node stoichiometry. As a consequence, only global duplications (e.g., WGD) are likely to succeed. For example, it is well-known that in yeast, polyploidy is at least initially better tolerated than aneuploidy [6,27,28].

However, why do the emerging pathways also seem to maximize the crosstalk with ancestral ones? This fact is independent from the previous, and constitutes another central finding of the analysis. The WGD created unusually many "cross-talk" links connecting duplicated pathways to each other and unusually few crosstalk-free linear pathways. The observation is motivated by the high occurrence of the ε-type duplication subgraph compared to the a pattern (Additional File 1, Table S2), but also by the high occurrence of side-by-side adjacent duplication subgraphs with β-β and γ-γ patterns (which are given in Fig. 5 and can be visualized in Additional File 1, Figure S2). The observation is also related to a previously reported enrichment of "bi-fan" arrays in the network of whole-genome duplicates [12]. One possible interpretation of this is that the emergent pathways largely encode for regulatory redundancy [5,23]. We can also hypothesize that this feature has to do with the fact that the transcriptional pathways emerging from the WGD emerged to regulate newly-formed metabolic pathways, to be used in alternative to the ancient ones [29,30], giving rise to the need for some sort of combinatorial cross-exclusion of the alternative metabolic genes to activate. The same hypothesis should predict enhanced combinatorial regulation of WGD duplicates, which could possibly be tested by a direct large-scale promoter sequence analysis. Further verification of the same hypothesis can be sought by combined analysis of metabolic and transcriptional plasticity after the yeast WGD. To our knowledge, previous observations are compatible with these speculations [11].

Finally, while in this work we have used the one-shot network duplication model mainly as a statistical tool, the simple patterns we have found can be used as the basis for a possible positive, or predictive, model. We have given a proof of principle of such a model, and we are currently exploring in more depth the possible phenomenology and implications, and the possibility of testing the predictions by analysing different whole-genome duplications.

## Abbreviations

WGD: whole-genome duplication; TF: transcription factor; DS: duplication subgraph;

## Authors' contributions

MCL designed and performed research, and wrote the paper. MC, BB, LG and DF designed and performed research. All authors read and approved the final manuscript.

## Supplementary Material

Additional file 1 **The file contains additional details and results on the statistical analysis and the spin model described in this work**. Click here for file
