# Supplementary material for: Ordered structure of the transcription network inherited from the yeast whole-genome duplication
Source: BMC Syst Biol. 2010 Jun 3;4:77. doi: 10.1186/1752-0509-4-77 (PMC2900227; doi:10.1186/1752-0509-4-77)
Supplement: Additional file 1 — The file contains additional details and results on the statistical analysis and the spin model described in this work. [file 1752-0509-4-77-S1.PDF]

Additional Text for Fusco *et al.*

Additional Section 1. SUBGRAPH-BASED MODEL

Statistically independent conservation of interactions vs duplication subgraphs. We provide here an argument in favor of a model based on duplication subgraphs. The simplest available model for the process under exam (See e.g. Presser *et al.* PNAS 105 2008, and Evlampiev and Isambert, PNAS 105 2008) would divide network evolution in two basic steps of duplication and divergence. Firstly, every node is duplicated, so that each ancestral interaction gives rise to four links. Secondly, several links are lost with a different independent probability associated to every link, mimicking the process of divergence. We can apply this mechanism to the directed transcription network of *S. cerevisiae*. Let us define the different probabilities of having conserved links between duplicate transcription factors TF_1 and TF_2 and duplicate targets tg_1 and tg_2 as p_{11} (related to the link $TF_1 \longrightarrow tg_1$), p_{12} (related to the link $TF_1 \longrightarrow tg_2$), p_{21} (related to the link $TF_2 \longrightarrow tg_1$) and p_{22} (related to the link $TF_2 \longrightarrow tg_2$).

Following the work of Evlampiev and Isambert, we assume that one link of the four is under selective pressure to be conserved in order to preserve the ancestral functionality. For simplicity, we identify this always with the same link and assume that p_{11} is equal to 1. As the probabilities are independent, it is possible to write a relationship between the probabilities of finding the different duplication subgraphs: P_α, P_β, P_γ, P_δ, P_ϵ, and P_ζ,

$$P_\alpha = p_{22}\,(1-p_{12})\,(1-p_{21}) \tag{1}$$

$$P_\beta = p_{12}\,(1-p_{21})\,(1-p_{22}) \tag{2}$$

$$P_\gamma = p_{21}\,(1-p_{12})\,(1-p_{22}) \tag{3}$$

$$P_\delta = p_{12}\,p_{21}\,(1-p_{22}) + p_{12}\,p_{22}\,(1-p_{21}) + p_{21}\,p_{22}\,(1-p_{12}) \tag{4}$$

$$P_\epsilon = p_{22}\,p_{12}\,p_{21} \tag{5}$$

$$P_\zeta = (1-p_{12})\,(1-p_{21})\,(1-p_{22}). \tag{6}$$

Using the observed frequency of the first three duplication subgraphs, one can solve the first three equations finding two solutions for the link probabilities:

Sol	p_{12}	p_{21}	p_{22}
1	0.0119	0.0438	0.0004
2	0.9345	0.9820	0.3471

The last equation for P_ζ excludes the second of these two solutions.

Given these probabilities, we can infer the probabilities associated to subgraphs of types δ and ϵ. We compared them with the observed frequencies of the subgraphs using a binomial distribution, obtaining a P-value smaller than 10^{-14}. The result suggests that, under these assumptions, the emerging interactions among duplicates cannot be considered independent.

Estimate for P_0. Our analysis allows to estimate the probability that an ancestral interaction is completely lost during evolution. Based on previous studies, it is possible to establish that 90% of the duplicated nodes was lost after the WGD event. If we focus on the leaves of the network, this implies that every node has a 0.9 chance to lose its duplicated sibling g. We can write the following relationship between this probability and the duplication subgraph probabilities estimated form their empirical occurrence

$$p(\text{loss } g) = (P_0 + P_\zeta/2 + P_\gamma/2)^{r_g} = \phi^{r_g} \ , \tag{7}$$

where r_g is the number of nodes with g incoming interactions.

Based on previous studies on transcription networks topology we can assume that the in-degree sequence follows a Poisson distribution, consequently we can compute the mean value of $p(\text{loss})$

$$p(\text{loss}) = 0.9 = 2\sum_k \phi^k e^{-\lambda}\frac{\lambda^k}{k!} = 2e^{\lambda(\phi-1)}. \tag{8}$$

Using the observed value of λ in the network, we estimate

$$P_0 = \frac{\phi - P_F^{emp}/2 - P_C^{emp}/2}{1 - P_F^{emp}/2 - P_C^{emp}/2} \simeq 0.34 \ . \tag{9}$$

Additional Section 2. ADDITIONAL RESULTS ON DUPLICATION SUBGRAPH OCCURRENCE

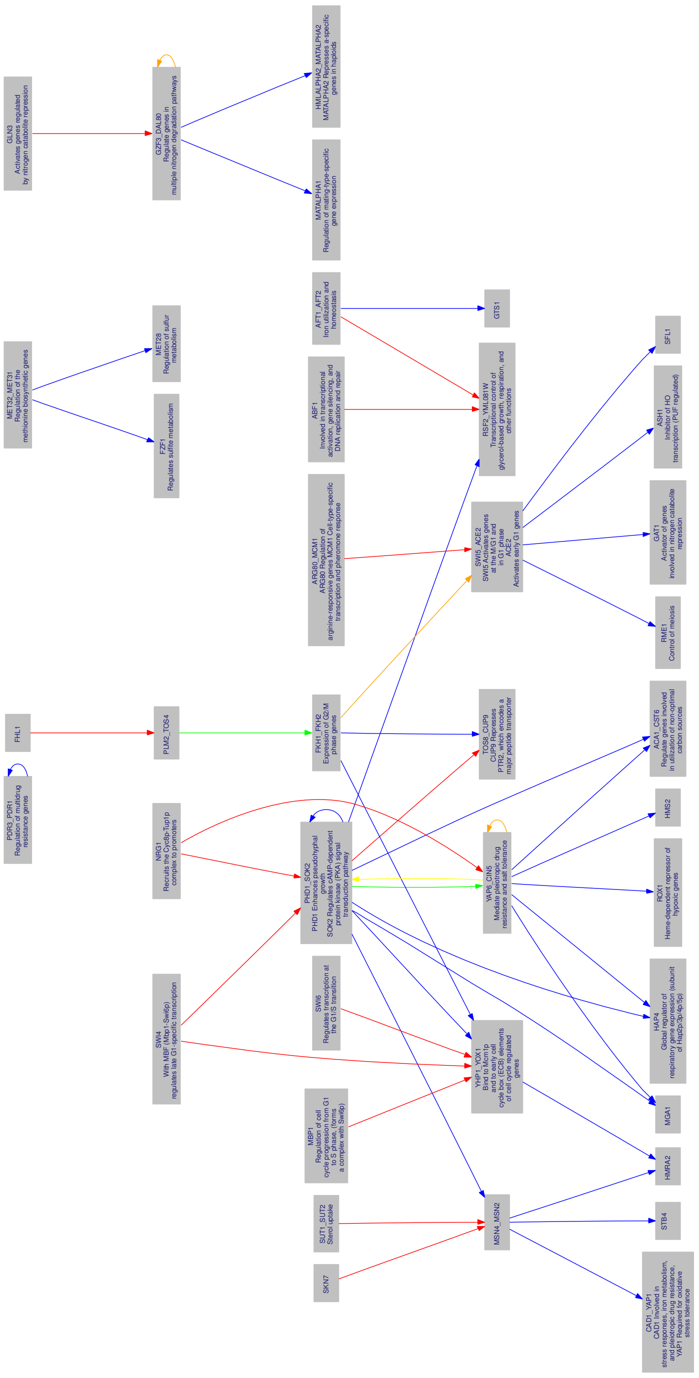

Additional Figure S1 The yeast transcription factor WGD network, formed by all 48 nontrivial DSs joining regulatory nodes (and having a connected component of 41 DSs). DSs are represented by colors following the conventions of the main text. The text within the 40 nodes of the graph gives functional annotations for the transcription factors and their duplicates from the SGD database (ftp://ftp.yeastgenome.org/yeast/ Feb 2009). The resulting interconnected pathways appear to involve many cell-cycle related transcription factors, but also pleiotropic drug resistance, respiration, DNA repair, sulfur, nitrogen and carbon metabolism, hypoxia.

The following tables contain a more detailed account of the results on DS occurrence already given in the main text. The lists of duplicates for this analysis were also integrated using the Yeast Gene Order Browser database 2.0 (Scannell *et al*, PNAS 104 20 2007), without any significant change in the results.

DUPLICATION SUBGRAPH OCCURRENCE								
Dupl. age class	α	β	γ	δ	ϵ	ζ	β/ζ	γ/ζ
A	0	38	0	0	0	42	0.905	0
B	0	9	0	0	0	20	0.450	0
C	0	3	0	0	0	52	0.058	0
D	0	5	0	0	0	95	0.053	0
E	0	38	0	0	0	371	0.102	0
all post-WGD	0	106	7	0	0	648	0.155	0.011
WGD	5	134	511	8	6	4906	0.027	0.104
G	0	32	3	0	0	296	0.108	0.010
H	0	9	1	9	9	149	0.060	0.007
I	0	26	11	0	0	1155	0.023	0.010
all pre-WGD	0	73	16	0	0	1610	0.045	0.010

TABLE S1 Occurrence of the different types of duplication subgraphs corresponding to the duplication age groups of Wapinski and coworkers. Post-WGD classes (A to E) are considered together on the sixth line of the table. Class A, the most recent, is the only one containing more β than ζ patterns, possibly because of incomplete divergence of interactions between recent duplicates. The figures for all pre-WGD duplications (G,H,I) are collected on the last line. The last two columns report the ratios β/ζ and γ/ζ related to non-shared interactions with duplicates. The first appears to decrease roughly with time and drop in the WGD, and the second ratio to be higher for the WGD network despite of the abundance of ζ pattern. See the duplication subgraph legend in Fig. 2.

WGD PARALOGS					
Dup.	Subgraph	Empirical value	Mean in Null Model	St. Dev.	Zscore
↓↓	α	8	6.984	3.329	+0.305
↙↘	β	193	35.01	6.454	+24.479
↘↙	γ	359	50.05	21.06	+14.67
↓↘↓	δ	12	0.707	0.9128	+11.225
↓⤫↓	ϵ	10	0.035	0.1944	+51.26

TABLE S2 Comparison between the empirical duplication subgraph occurrence and a null model shuffling homology classes for WGD paralogs. All the subgraphs excluding type α are overrepresented. See the duplication subgraph legend in Fig. 2.

NON-WGD PARALOGS

Dup.	Subgraph	Empirical value	Mean in Null Model	St. Dev.	Zscore
$\downarrow\downarrow$	α	4983	7847	1351	-2.120
$\swarrow\searrow$	β	24269	33380	4372	-2.084
$\searrow\swarrow$	γ	15413	15380	2853	+0.012
$\downarrow\searrow\downarrow$	δ	1119	949.8	232.6	+0.727
$\downarrow\searrow\!\!\!\!\!\swarrow\downarrow$	ϵ	166	35.18	17.64	+7.416

TABLE S3 Comparison between the empirical duplication subgraph occurrence and a null model shuffling homology classes for non-WGD paralogs. The only overrepresented subgraph is ϵ. Subgraphs α and β are underrepresented.

PRE-WGD PARALOGS

Dup.	Subgraph	Empirical value	Mean in Null Model	St. Dev.	Zscore
$\downarrow\downarrow$	α	0	6.19	12.48	-0.59
$\swarrow\searrow$	β	73	6.16	10.43	+6.41
$\searrow\swarrow$	γ	16	6.11	10.40	+0.95
$\downarrow\searrow\downarrow$	δ	0	6.24	10.61	-0.59
$\downarrow\searrow\!\!\!\!\!\swarrow\downarrow$	ϵ	0	5.94	10.13	-0.59

TABLE S4 Comparison between the empirical duplication subgraph occurrence and a null model shuffling homology classes for pre-WGD paralogs. The result for subgraph β (duplication of target) changes compared to the analysis that groups all non-wgd DSs. In addition, ϵ-type DSs are not overrepresented in this analysis.

POST-WGD PARALOGS

Dup.	Subgraph	Empirical value	Mean in Null Model	St. Dev.	Zscore
$\downarrow\downarrow$	α	0	3.59	7.41	-0.48
$\swarrow\searrow$	β	106	3.86	7.84	+13.03
$\searrow\swarrow$	γ	7	3.81	7.84	+0.41
$\downarrow\searrow\downarrow$	δ	0	3.79	7.91	-0.48
$\downarrow\searrow\!\!\!\!\!\swarrow\downarrow$	ϵ	0	3.84	7.85	-0.49

TABLE S5 Comparison between the empirical duplication subgraph occurrence and a null model shuffling homology classes for post-WGD paralogs. The behaviour of subgraph β (duplication of target) changes completely compared to the previous analysis. In addition, subgraph ϵ is not overrepresented in this analysis.

Additional Section 3. ADDITIONAL RESULTS ON NEIGHBOR DUPLICATION SUBGRAPH CORRELATION

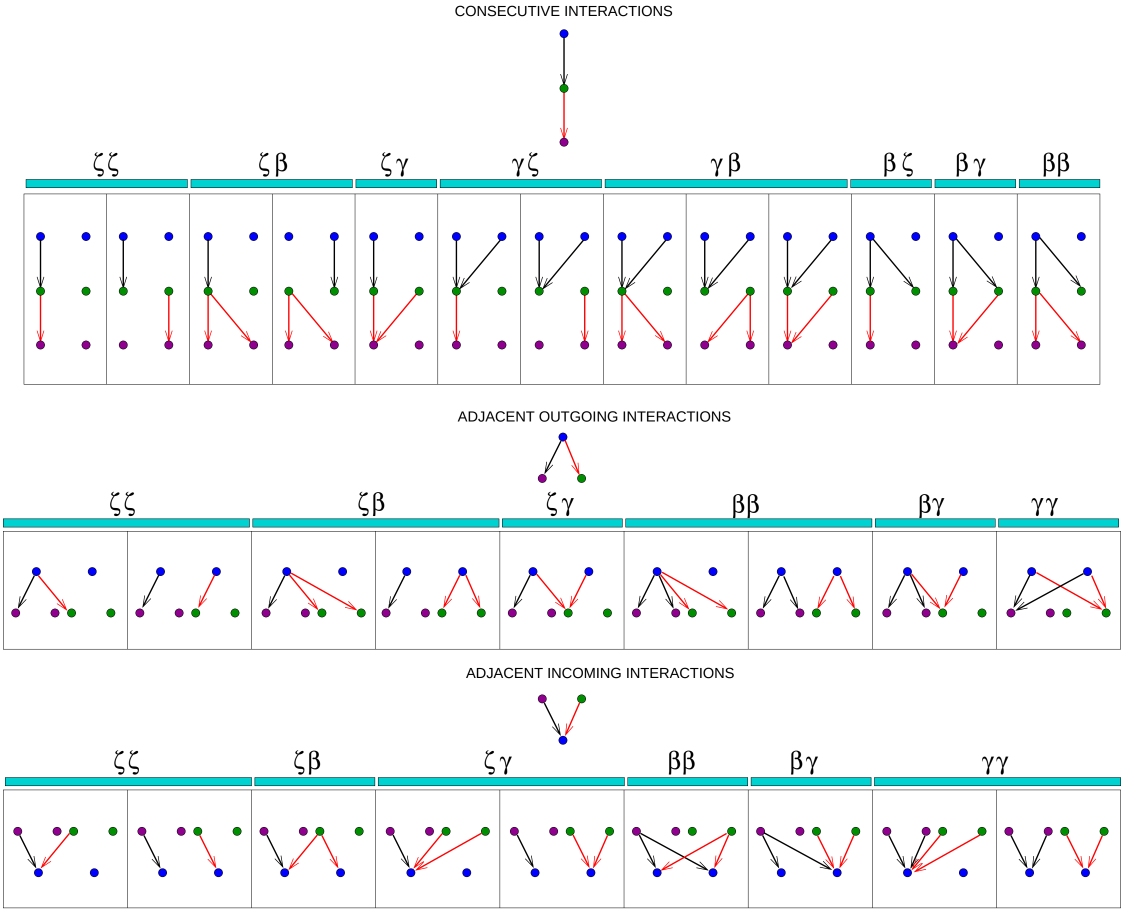

Additional Figure S2 All the possible interactions among duplication subgraphs of the kinds ζ, β and γ, including the possibility of connected and non-connected resulting DSs. Duplication subgraphs are divided depending on the kind of relationnship between neighbor ancestral links: conscutive, adjacent outgoing and adjacent incoming. See the duplication subgraph legend in Fig. 2 of the main text.

The following tables contain a more detailed account of the results on DS correlation already given in the main text.

CONSECUTIVE INTERACTIONS						
DS1	DS2	Connected	Emp. Occurrence	Mean in Model	St. dev.	Zscore
ζ	ζ	yes	33595	24390.1	409.773	22.4634
ζ	ζ	no	10414	24398.9	428.694	-32.6221
ζ	β	yes	356	281.046	32.017	2.34107
ζ	β	no	55	282.811	32.8684	-6.93101
ζ	γ	yes	2792	2158.66	116.743	5.42511
β	ζ	yes	3531	588.377	368.163	7.99271
β	β	yes	9	6.751	5.11283	0.439874
β	γ	yes	527	25.939	16.6818	30.0363
γ	ζ	yes	1625	1075.6	346.276	1.58659
γ	ζ	no	1254	1075.12	345.783	0.517324
γ	β	yes	6	12.516	5.54597	-1.17491
γ	β	no	7	12.272	5.48562	-0.961058
γ	γ	yes	232	94.886	33.0569	4.14781

TABLE S6 Comparison between the empirical values of neighbour duplication subgraphs for consecutive interactions with the distribution generated by the one-shot duplication null model. The annotation of interactons follows Supplementary Fig. S2, distinguishing between connected and non-connected DSs. See the duplication subgraph legend in Fig. 2.

ADJACENT OUTGOING INTERACTIONS						
DS 1	DS 2	Connected	Emp. Ocurrence	Mean in Model	St. dev.	Zscore
ζ	ζ	yes	855020	562225	2972.3	98.5082
ζ	ζ	no	239769	562243	2960.58	-108.923
ζ	β	yes	22689	13033.2	1322.71	7.30005
ζ	β	no	2372	13033.9	1314.7	-8.10976
ζ	γ	yes	117829	99512.1	4981.65	3.67686
β	β	yes	675	75.606	17.6526	33.9551
β	β	no	9	75.766	17.7407	-3.76344
β	γ	yes	581	1154.76	137.618	-4.16922
γ	γ	yes	14612	2202.95	236.127	52.5524

TABLE S7 Comparison between the empirical values of neighbour duplication subgraphs for adjacent outgoing interactions with the distribution generated by the one-shot duplication null model. The annotation of interactons follows Supplementary Fig. S2, distinguishing between connected and non-connected DSs.

ADJACENT INCOMING INTERACTIONS						
DS 1	DS 2	Connected	Emp. Occurrence	Mean in Model	St. dev.	Zscore
ζ	ζ	yes	22806	12517.9	116.528	88.2882
ζ	ζ	no	1345	12522.3	110.145	-101.479
ζ	β	yes	645	584.177	73.7217	0.825035
ζ	γ	yes	2811	1111.57	74.8757	22.6967
ζ	γ	no	141	1110.78	74.6193	-12.9964
β	β	yes	67	3.451	2.00888	31.634
β	γ	yes	35	26.329	6.07756	1.42672
γ	γ	yes	74	24.739	5.66894	8.68963
γ	γ	no	2	24.715	5.86309	-3.87424

TABLE S8 Comparison between the empirical values of neighbour duplication subgraphs for adjacent incoming interactions with the distribution generated by the one-shot duplication null model. The annotation of interactons follows Supplementary Fig. S2, distinguishing between connected and non-connected DSs.

Additional Section 4. SIMPLIFIED POSITIVE ONE-SHOT DUPLICATION MODEL. DEFINITION AND MONTE CARLO SIMULATIONS

In this section, we give some technical details on the simplified Monte Carlo model we adopted.

The Hamiltonian we considered can be written as

$$H = c \sum_{m \to n} \left(x_n^2 y_m - x_n y_m^2 \right) + d \sum_{m \leftrightarrow n} (x_n y_m) + \mu_1 \sum_n x_n + \mu_2 \sum_n x_n^2 \ , \tag{10}$$

where n e m are indices for network links, and x_n e y_m are spins representing the DSs on the links. These are discrete variables that take values in $\{0, \pm 1\}$, 0 represents ζ-type DSs, 1 represents β-type DSs, and -1 represents γ-type DSs. The sums $m \to n$ and $m \leftrightarrow n$ are carried over the pairs of consecutive and adjacent interactions (the two kinds are unified). Thus, the coupling constant c represents the interactions between conesutive DSs, while the coupling constant d represents interactions between adjacent DSs. The external fields μ_1 and μ_2 represent the cost for creating a link of different colors. These parameters are fixed *a priori* to reproduce the empirical occurrence of DSs. In other words, this simplified model does not consider the occurrence of DSs as an emergent property of their interactions. The model was simulated on the empirical collapsed pre-WGD network, using a Metropolis algorithm with single-spin flip moves. After thermalization with different parameter sets, the average occurrences and correlations in the simulation was compared to the empirical case.

Table S9 compares the $c = 0, d = 0$ case, which corresponds to the null model used in the main text, with the parameter set with $c < 0$ and $d < 0$ giving the best agreement with empirical data. It can be observed that, despite the extreme simplicity of the model, the empirical qualitative trends of most of the observables are obtained. While a full quantitative understanding of the minimal model and set of coupling constants required to establish the empirical trends remains an open issue, we consider these results a proof of principle that the methodology is a valid and promising approach.

DS occurrence			
DS	Frequency Empirical	Frequency Monte Carlo $c = 0, d = 0$	Frequency Monte Carlo $c = -0.06, d = -0.005$
ζ	94.66%	94.68%	94.25%
β	1.11%	1.10%	1.14%
γ	4.23%	4.23%	4.61%

Adjacent DS pairs			
DS	Frequency Empirical	Frequency Monte Carlo $c = 0, d = 0$	Frequency Monte Carlo $c = -0.06, d = -0.005$
$\zeta \longleftrightarrow \zeta$	87.33%	89.47%	88.42%
$\zeta \longleftrightarrow \beta$	2.01%	2.16%	2.06%
$\zeta \longleftrightarrow \gamma$	9.40%	7.98%	9.05%
$\beta \longleftrightarrow \beta$	0.06%	0.035%	0.034%
$\beta \longleftrightarrow \gamma$	0.048%	0.099%	0.11%
$\gamma \longleftrightarrow \gamma$	1.15%	0.26%	0.34%

Consecutive DS pairs			
DS	Frequency Empirical	Frequency Monte Carlo $c = 0, d = 0$	Frequency Monte Carlo $c = -0.06, d = -0.005$
$\zeta \longrightarrow \zeta$	80.92%	90.27%	83.85%
$\zeta \longrightarrow \beta$	0.77%	1.09%	1.0%
$\zeta \longrightarrow \gamma$	4.69%	4.09%	4.14%
$\beta \longrightarrow \zeta$	6.67%	0.57%	7.22%
$\beta \longrightarrow \beta$	0.017%	0.0085%	0.083%
$\beta \longrightarrow \gamma$	1.00%	0.025%	0.53%
$\gamma \longrightarrow \zeta$	5.46%	3.73%	3.0%
$\gamma \longrightarrow \beta$	0.025%	0.045%	0.032%
$\gamma \longrightarrow \gamma$	0.44%	0.17%	0.15%

TABLE S9 Resume of the results of the Monte Carlo simulations comparing the null model with a model with interaction constants between neighbor DSs. At equal magnetizations, the appearence of interactions tends to bring the equilibrium network configurations closer to the empirical case.
